# Impact of Vitamin D on the Cardiovascular System in Advanced Chronic Kidney Disease (CKD) and Dialysis Patients

**DOI:** 10.3390/nu10060709

**Published:** 2018-06-01

**Authors:** Anna Gluba-Brzózka, Beata Franczyk, Aleksandra Ciałkowska-Rysz, Robert Olszewski, Jacek Rysz

**Affiliations:** 1 Department of Nephrology, Hypertension and Family Medicine, WAM Teaching Hospital, 90-549 Lodz, Poland; aniagluba@yahoo.pl; 2 Department of Nephrology, Hypertension and Family Medicine, Medical University of Lodz, 90-549 Lodz, Poland; bfranczyk-skora@wp.pl (B.F.); jacek.rysz@umed.lodz.pl (J.R.); 3 Palliative Care Ward, Department of Oncology, Medical University of Lodz, 90-549 Lodz, Poland; olarysz@rmed.pl; 4 Department of Ultrasound, Institute of Fundamental Technological Research, Polish Academy of Sciences (IPPT PAN), 02-106 Warsaw, Poland; robert.olszewski@me.com

**Keywords:** vitamin D, chronic kidney disease, cardiovascular disease, mortality, vitamin D analogues, treatment

## Abstract

In patients suffering from chronic kidney disease (CKD), the prevalence of cardiovascular disease is much more common than in the general population. The role of vitamin D deficiency had been underestimated until a significant association was found between vitamin D therapy and survival benefit in haemodialysis patients. Vitamin D deficiency is present even in the early stages of chronic kidney disease. The results of experimental studies have revealed the relationship between vitamin D deficiency and impairment of cardiac contractile function, higher cardiac mass and increased myocardial collagen content. Experimental models propose that intermediate end points for the relationship between vitamin D deficiency and higher risk of cardiovascular disease comprise diminished left ventricular hypertrophy (LVH), enhanced left ventricular diastolic function, and decreased frequency of heart failure. Multiple observational studies have demonstrated an association between the use of active vitamin D therapy in patients on dialysis and with CKD and improved survival. However, there are also many studies indicating important adverse effects of such treatment. Therefore, large randomized trials are required to analyze whether supplementation of vitamin D may affect outcomes and whether it is safe to be used in CKD patients.

## 1. Introduction

In patients suffering from chronic kidney disease (CKD), the prevalence of cardiovascular disease is much more common than in the general population [1]. Their high morbidity and mortality cannot be explained by traditional cardiovascular risk factors, such as advanced age, the presence of diabetes, hypertension, hypertriglyceridemia and low levels of high-density lipoprotein (HDL) cholesterol [2]. According to studies, also abnormalities of calcium, phosphorus, vitamin D, and parathyroid hormone (PTH) are associated with the occurrence of cardiovascular disease [3,4,5]. Some of them also indicate a relationship between vitamin D deficiency and hypertension, insulin resistance, diabetes, and dyslipidaemia [6,7,8,9]. Vitamin D deficiency is present even in the early stages of chronic kidney disease. Numerous observational studies have confirmed low levels of both total 25-hydroxyvitamin D 25(OH)D which enables the assessment of the adequacy of vitamin D stores) and 1,25-dihydroxyvitamin D (1,25(OH)2D—biologically active form of vitamin D), in patients with CKD and end-stage renal disease (ESRD) [10,11].

The role of vitamin D deficiency had been underestimated until a significant association was found between vitamin D therapy and survival benefit in haemodialysis patients.

Cascade of changes in mineral metabolism occur in the course of chronic kidney disease resulting in the increase in the level of circulating parathyroid hormone and in abnormalities associated with secondary hyperparathyroidism (SHPT). In consequence, higher morbidity and mortality have been observed in patients with CKD stages 3–5D [5,12]. Management of elevated PTH levels in CKD has posed a challenge for several decades.

Patients with chronic kidney disease are frequently suffering from the deficiency of 1,25-dihydroxyvitamin D3 (calcitriol) due to the lack of its precursor–25-hydroxyvitamin D3, and also due to the decreased activity of kidney enzyme 1α-hydroxylase, which converts this precursor into active hormone [13,14].

The recommended dietary levels (the requirement of over 97.5% of the population) for vitamin D are 600 IU/d for individuals aged 1–70 years and 800 IU/d for those older than 70 years [15]. In the opinion of Institute of Medicine Committee, evidences that vitamin D prevented cardiovascular disease, diabetes, or other cardiometabolic outcomes are inconsistent and inconclusive [16]. Moreover, they do not meet criteria for establishing a cause–effect relationship.

Low levels of 25(OH)D in patients with kidney disease may be due to the loss of vitamin D binding protein in the urine [17], defective photoproduction in the skin following the exposure to ultraviolet B radiation [18], and likely reduced nutritional intake and sun exposure [19].

## 2. Vitamin D and Its Deficiency

Vitamin D plays an important role in calcium–phosphorus homeostasis, regulation of PTH, and formation and maintenance of bones [3]. Two distinct forms of vitamin D exist—vitamin D2 (ergosterol-derived molecule) and vitamin D3 (cholesterol-derived molecule) [20]. 7-Dehydrocholesterol (pro-vitamin D3) and cholecalciferol (pre-vitamin D3) are precursors of vitamin D3, while ergocalciferol (pre-vitamin D2) is the precursor of vitamin D2. Both vitamin D2 and D3, irrespective of vitamin D source (ultraviolet B-induced synthesis in the skin or nutritional intake), are hydroxylated to 25-hydroxyvitamin D [25(OH)D] (calcidiol) in the liver and then to biologically active form 1,25-dihydroxyvitamin D (1,25[OH]2D; calcitriol) [21]. The concentration of circulating 25(OH)D is 1000-fold higher than that of most potent vitamin D metabolite 1,25-dihydroxyvitamin D (1,25(OH)2D; calcitriol) [22,23,24]. Renal production of 1,25(OH)2D is strictly controlled by homoeostatic mechanisms, however, it becomes considerably dependent on substrate availability when the level of circulating 25(OH)D is low [22,23]. According to studies, also myocardium and vasculature express 1α-hydroxylase and therefore, they can also be the source of 1,25(OH)2D [22,24].

It is estimated that in nearly 1/3–1/2 of healthy middle-aged to elderly adults, levels of 25-hydroxy vitamin D [25(OH) D] which is the principal circulating storage form of vitamin D, are low [4,25]. Vitamin D insufficiency is usually diagnosed in adults and children when its level drop below 75 nmol/L (30 ng/L), while deficiency is identified in case of vitamin D level below 50 nmol/L (20 ng/L) [4,22,26]. In the course of CKD, renal calcitriol synthesis is diminished as a result of counter-regulatory effects of increasing fibroblast growth factor 23 (FGF23) levels as well as of loss of renal parenchyma [5,27]. Both reduced calcitriol levels and hypocalcaemia stimulate the synthesis PTH. The severity of secondary hyperparathyroidism aggravates with worsening renal function leading to parathyroid cell proliferation and hyperplasia as well as the occurrence of abnormalities in bone mineralisation and turnover. Calcitriol or vitamin D receptor activator (VDRA) treatment including paricalcitol, doxercalciferol and alfacalcidol is associated with the suppression of PTH levels, but, at the same time it can lead to the rise in serum calcium, phosphate and FGF23 levels [5]. This inhibition is the result of calcitriol/VDRA action through the vitamin D receptor (VDR) [5].

Most effects associated with vitamin D deficiency relate to musculoskeletal system, however, numerous studies point that it adversely affects also cardiovascular system [28]. Vitamin D was suggested to protect against cardiovascular disease, however, the observed effects of vitamin D on CKD patients’ outcomes are controversial.

Cardiac cells contractile properties are controlled by direct interaction between calcium, contractile proteins, actin and myosin, and the intracellular handling of calcium [4]. Due to the fact that vitamin D level influences extracellular calcium homeostasis, it alters intracellular calcium thus indirectly affecting cardiac cell contractility [29]. Moreover, active form of vitamin D (1,25-dihydroxyvitamin D) exerts impact also on endothelial cells, and vascular smooth muscle cells through the cytosolic vitamin D receptor (VDR). It affects morphology, proliferation and growth of cardiac cells [4]. According to studies, the therapy with calcitriol enhances the expression and nuclear localization of VDR receptors, boosts cardiac muscle protein expression and diminishes the expression of atrial natriuretic peptides (ANPs) [4,30]. Vitamin D has been shown to modulate the expression of tissue matrix metalloproteinases (MMPs) secreted by activated macrophages during inflammatory responses, thus protecting against the development of atherosclerosis [31]. According to studies, calcitriol is of key importance not only for calcium homeostasis, but also for the homeostasis of electrolytes, volume, and blood pressure [4].

Vast majority of haemodialysis patients suffer from vitamin D deficiency [23,32]. According to studies, either relative or absolute vitamin D deficiency of 25(OH)D in patients with CKD and ESRD as well as in general population have been associated with all-cause mortality, cardiovascular events, peripheral vascular disease, congestive heart failure, hypertension and the later need for renal replacement therapy [19,23,33,34,35,36]. The results of experimental studies have revealed the relationship between vitamin D deficiency and impairment of cardiac contractile function [37], higher cardiac mass [38] and increased myocardial collagen content [39]. Experimental models propose that intermediate end points for the relationship between vitamin D deficiency and higher risk of cardiovascular disease comprise diminished left ventricular hypertrophy (LVH), enhanced left ventricular diastolic function, and decreased frequency of heart failure [40,41]. Cardiomyocyte-specific deletion of the vitamin D receptor gene resulted in cardiac hypertrophy and increased left ventricle (LV) weight [42]. In turn, activated vitamin D therapy was associated with decreased myocardial hypertrophy in experimental models of cardiac hypertrophy [43] and prevented the development of heart failure [44]. Moreover, it has been suggested that vitamin D supplementation may protect cardiovascular health through modulation of the renin-angiotensin system [3]. Li et al. [45,46] suggested that vitamin D was a negative endocrine regulator of renin biosynthesis in vivo, since renin mRNA and protein levels in the kidney of both vitamin D receptor knockout mice and 25-hydroxyvitamin D1α-hydroxylase knockout mice were found to be significantly increased. Moreover, it has been observed that vitamin D receptor knockout mice developed hypertension and cardiac hypertrophy as a result of renin-angiotensin system dysregulation [45,47]. The results of numerous studies confirmed that 1,25-dihydroxyvitamin D3 directly suppressed renin gene expression, and this effect was independent of vitamin D influence on calcium metabolism.

The fact that low vitamin D concentrations relate with altered myocardial calcium flux and increased risk of sudden cardiac death (SCD) may suggest its link to cardiac arrhythmias [48,49]. The finding that calcitriol therapy reduced prolonged QT interval (the time between the start of Q wave and the end of T wave) (risk factor for SCD) in haemodialysis patients is in agreement with the previous suggestion [50,51].

Studies on animal models indicated a relationship between vitamin D3 deficiency and considerable hypocalcaemia, rise in plasma parathyroid hormone level and accumulation of collagen fibres leading to interstitial fibrosis in rats [37]. It seems that the relationship between vitamin D deficiency and cardiovascular disease involve not only atherosclerosis but also vascular calcification. Some studies suggest that excess vitamin D contributes to risk of hypercalcemia and vascular calcification leading to increased survival and morbidity [52,53]. In patients with end-stage renal disease, in whom renal calcitriol synthesis is diminished, secondary hyperparathyroidism is highly prevalent [54] and it is the primary cause of cardiovascular disease.

**Figure 1:**
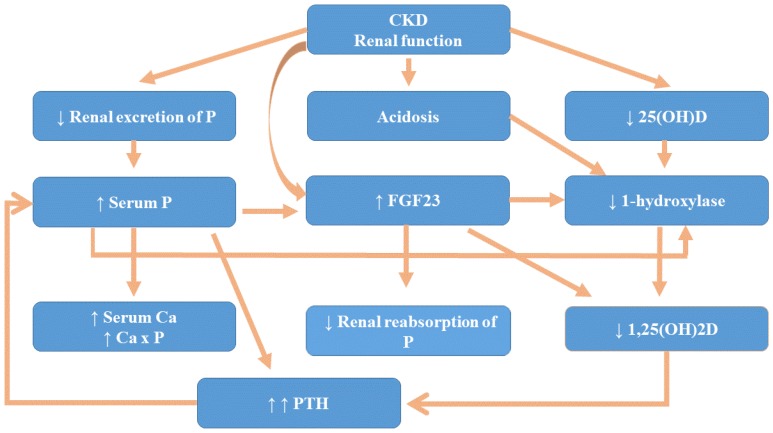
Mechanism contributing to progressive decrease in 1,25(OH)2_2_D in patients with Chronic Kidney Disease (CKD). FGF23: fibroblast growth factor 23; PTH: parathyroid hormone.

Figure 1 summarizes the mechanism contributing to progressive decrease in 1,25(OH)2D in patients with CKD.

## 3. Vitamin D Deficiency Treatment

In patients with CKD, several analogues of vitamin D have been studied. Paricalcitol and doxercalciferol are D2, while calcitriol is a D3 compound [6]. Doxercalciferol is an inactive pro-hormone which requires the conversion by the liver to its active form (1,25-dihydroxyvitamin D2).

Animal studies, demonstrated that calcitriol and related analogues including paricalcitol enhanced diastolic relaxation and decreased end-diastolic pressures, lowered cardiac mRNA expression and blood levels of natriuretic peptides, and diminished the frequency of congestive heart failure episodes [38,41,48]. Moreover, supplementation of active vitamin D as well as synthetic vitamin D analogues has been demonstrated to lower the risk of mortality of cardiovascular causes [55] and it inversely correlated with the extent of vascular calcification, independently from other risk factors for ischemic heart disease [56]. Numerous studies have demonstrated decreased mortality in populations of dialysis patients treated with ‘active’ vitamin D, either calcitriol or vitamin D receptor activators [57,58].

A prospective, randomized, placebo-controlled trial [39] examining the hypothesis that treatment with oral activated vitamin D (paricalcitol), decreases LV mass in stages 3–5 CKD patients with LV hypertrophy and that it improves systolic and diastolic dysfunction in CKD, demonstrated that 52 weeks of treatment with paricalcitol (at a dose that is sufficient to suppress secondary hyperparathyroidism), failed to reduce LV mass and volume in CKD stages 3–5 patients with LV hypertrophy and it did not modify LV systolic function nor diastolic function. Also, in this study paricalcitol treatment was associated with hypercalcaemia, which occurred in 43.3% of participants. A meta-analysis performed by Li et al. [59] analysing effects of active vitamin D on cardiovascular outcomes in predialysis patients with CKD demonstrated that calcitriol/VDRA therapy was associated with a reduced incidence of cardiovascular events (relative ratio (RR) 0.27 (95% confidence interval (CI) 0.13–0.59)) and reduced proteinuria (RR 1.9 (95% CI 1.34–2.71) in comparison to placebo or no treatment. In this study, paricalcitrol administration, but not calcitriol therapy, increased probability of hypercalcaemia with RR 7.85 (95% CI 2.92–21.10). The hypercalcaemic risk with calcitriol/VDRA is one of the principal concerns with this therapy in CKD, which raises safety concerns. The treatment of severe and progressive SHPT should begin with low doses of calcitriol/VDRA, independent of the initial PTH concentration, and it should be further titrated on the basis of PTH response, to avoid hypercalcaemia.

The meta-analysis of 20 observational studies (11 prospective cohorts, 6 historical cohorts and 3 retrospective cohorts) [7] demonstrated that the therapy with various analogues of active vitamin D (paricalcitol, calcitriol) was associated with differences in patients’ survival. It turned out that treatment with paricalcitol lowered mortality to a slightly greater extent than calcitriol therapy, which could be explained by their differential effects on vascular calcification. The administration of vitamin D was associated with lower mortality compared to those with no treatment (adjusted case mixed baseline model: hazard ratio (HR), 0.74; 95% confidence interval (95% CI), 0.67–0.82; *p* <0.001; time-dependent Cox model: HR, 0.71; 95% CI, 0.57–0.89; *p* < 0.001). Analysis of calcitriol and paricalcitrol separately also provided significant decrease in CAD mortality (HR, 0.63; 95% CI, 0.50–0.79; *p* < 0.001) and (HR, 0.43 95% CI, 0.29–0.63; *p* < 0.001), respectively. It has been observed that patients receiving paricalcitol had a survival advantage over those who received calcitriol (HR, 0.95; 95% CI, 0.91–0.99; *p* < 0.001). Alfacalcidol, calcitriol, paricalcitol and not otherwise specified active vitamin D treated patients had a 46% (HR, 0.54; 95% CI, 0.37–0.80), 43% (HR, 0.57; 95% CI, 0.46–0.70), 27% (HR, 0.73; 95% CI, 0.62–0.87) and 36% (HR, 0.64; 95% CI, 0.57–0.72) lower overall mortality risk in comparison to untreated patients. Moreover, with calcitriol, paricalcitol and not otherwise specified active vitamin D supplementation resulted in 26% decrease (HR, 0.74; 95% CI, 0.55–0.99), 39% (HR, 0.61; 95% CI, 0.58–0.64) and 30% (HR, 70; 95% CI, 0.63–0.79) in all-cause mortality risk compared to persons without active vitamin D treatment. The survival advantage was similar in groups of ESRD on dialysis patients and CKD patients not on dialysis. According to other studies, vitamin D (or its analogues – paricalcitol, calcitriol) administration to haemodialysis patients is associated with significant reduction in the risk of all-cause death and of cardiovascular death. The difference in survival of patients receiving paricalcitol was significant at 12 months and increased with time (*p* < 0.001) [60]. The effect of vitamin D analogues therapy may also depend on mean daily or weekly doses. In haemodialysis patients, weekly doses of paricalcitol above 15.0 μg were shown to result in an 18% reduction of mortality risk [61]. Too low doses of vitamin D exerts weak anti-vascular calcification effects in CKD patients, however, its high doses of could be associated with the occurrence of adverse effects, including hypercalcemia, which would overwhelm protective effects.

Shoji et al. [55] study demonstrated that the risk of death was lower in haemodialysis patients treated with alfacalcidol (vitamin D analogue) in comparison to persons not treated with this vitamin. In the study of Drechsler et al. [23] vitamin D status was found to be strongly associated with the risk of SCD. The unadjusted hazard to experience SCD was in haemodialysis patients with severe vitamin D deficiency 3-fold higher in comparison to those with sufficient 25(OH)D levels (HR: 2.99, 95% CI: 1.39–6.40). The adjustment for potential confounders and seasonal variation of 25(OH)D as well as markers of mineral metabolism including PTH, calcium, and phosphate did not influence the association (HR: 2.95, 95% CI: 1.35–6.46) and (HR: 3.00, 95% CI: 1.36–6.60), respectively. The analysis, in which 25(OH)D was treated as a continuous variable demonstrated even higher hazard to die suddenly (59% per unit decrease in 25(OH)D levels) [23]. In this study, the risk of stroke was higher in HD patients with lower 25(OH)D levels. This risk increased by 30% per unit decrease in 25(OH)D. However, the study of Drechsler et al. [23] failed to demonstrate any association between vitamin D status with MI.

Moreover, some studies indicate that activated vitamin D suppresses renin expression [45] and triggers apoptosis [62]. Administration of injectable vitamin D was shown to increase 2-year survival in comparison to patients who never received it (75.6% vs. 58.7%, *p* < 0.001) and to reduce mortality by 20% (HR, 0.80; 95% [CI], 0.76 to 0.83) [57]. In patients with serum level of PTH > 300 pg/mL (>33.0 pmol/L), the survival advantage for the injectable vitamin D group was 18% (HR, 0.82; 95% CI, 0.77 to 0.86) [57]. Multinational, double-blind, randomized placebo-controlled trial PRIOMO (Paricalcitol Capsule Benefits in Renal Failure—Induced Cardiac Morbidity) conducted in 11 countries and involving 227 patients with chronic kidney disease, mild to moderate left ventricular hypertrophy, and preserved left ventricular ejection fraction, analysed whether 48-week treatment with paricalcitol (19-nor-1,25-(OH)2 vitamin D2) reduced left ventricular mass, enhanced diastolic function, diminished CVD events, and improved the level of cardiac biomarkers in patients with LVH and CKD [14]. In this study, paricalcitol at dose 2 μg/d did not reduce left ventricle mass index (LVMI) and did not alter certain echocardiographic measures of diastolic function. However, it did reduce CVD hospitalizations and mitigate the increase in blood levels of BNP, especially in those with evident LVH at baseline. Despite being well-tolerated, paricalcitol treatment was in some patients (20.9%) associated with hypercalcemia. Moreover, paricalcitol also increased serum creatinine and consequently reduced creatinine-based measures of estimated glomerular filtration rate (eGFR) [14].

However, the effectiveness of calcitriol/VDRA in the CKD stage 3–4 population has been questioned and the use of the aforementioned compounds have been suspected to be associated with adverse effects, including hypercalcaemia and elevation in fibroblast growth factor 23 (FGF23) levels [5]. According to studies, calcitriol and vitamin D analogues decrease PTH (−196 pg/mL [95% CI, −298 to −94] in dialysis patients and (−49 pg/mL [95% CI, −86 to −13]) in pre-dialysis patients) but at the same time they increase serum phosphate and calcium levels [11,19,63].

## 4. Mechanisms Associated with Vitamin D Status

**Figure 2:**
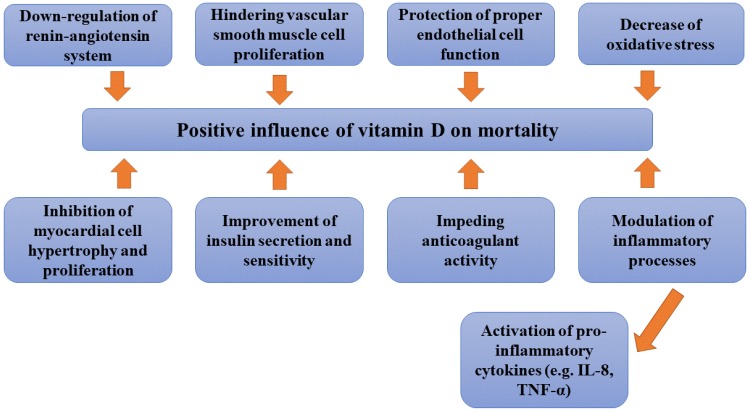
Mechanisms involved in the effect of vitamin D on altered mortality.

Vitamin D therapy decreases CAD risk due to multi-organ protective effects. There are at least several mechanisms involved in the effect of vitamin D on altered mortality [7] (Figure 2). The influence of vitamin D on mortality was suggested to be related with several mechanisms, including the down-regulation of renin-angiotensin system [64,65], protection of proper endothelial cell function [3], hindering vascular smooth-muscle cell proliferation [66], modulation of inflammatory processes [67], including the activation of pro-inflammatory cytokines as interleukin (IL)-8 and tumour necrosis factor (TNF)-alpha [68] and oxidative stress [69], impeding anticoagulant activity [70], inhibition of myocardial cell hypertrophy and proliferation [71] and finally the improvement of insulin secretion and sensitivity [72]. Despite the fact that the exact mechanism of CKD patient mortality has not been resolved, studies indicate that it can be associated with over vascular calcifications, left ventricle hypertrophy and in consequence left ventricle dysfunction [7]. Increased mortality risk in patients with low 25(OH)D levels may be the consequence of the relationship between vitamin D deficiency with cardiovascular risk factors, such as: type 2 diabetes mellitus [73], arterial hypertension [74], malnutrition, and inflammation [23]. In a double-blind, randomized, placebo-controlled trial, vitamin D supressed pro-inflammatory state via the down-regulation of nuclear factor-κB activity, hampering of the production of IL-6, IL-12, interferon-, and TNF while increasing that of anti-inflammatory cytokines [75]. Timms et al. [31] suggested that vitamin D administration in case of its deficiency may inhibit several aspects of the inflammatory response to cardiovascular injury as well as slow down atherosclerotic plaque progression and limit plaque rupture. Moreover, inverse correlation has been observed between serum vitamin D levels and coronary calcification [56]. Vitamin D may indirectly prevent inflammation and the stimulation of endothelial progenitor cells (EPCs) and in this way it protects against the development of congestive heart failure [76]. Experiments on animal models (rats) indicated that vitamin D deficiency promoted myocardial hypertrophy, greater heart weight/body weight ratio and extracellular matrix production in myocardial tissue in rats, while administration of 1,25-dihydroxyvitamin D3 inhibited cell proliferation of primary ventricular myocytes [37,77]. These observations were confirmed in haemodialysis patients, in whom vitamin D therapy (calcitriol) was associated with the reduction of left ventricle hypertrophy (LVH) accompanied by a considerable decrease in plasma renin activity and plasma angiotensin II levels [78]. The finding that 1,25-dihydroxyvitamin D3 hampered the expression of a skeletal actin and ANP gene (both associated with myocardial hypertrophy and heart failure) suggest its role in cardiac homeostasis [79]. The results of Multi-Ethnic Study of Atherosclerosis [80] suggest that vitamin D deficiency is prospectively associated with elevated risk of coronary artery calcification (a measure of coronary atherosclerosis). The association of 25-hydroxyvitamin D with incident CAC was observed to be stronger among participants with lower estimated GFR. Overt association of vitamin D deficiency with SCD but not with MI may suggest that atherosclerosis related to vitamin D deficiency might not be the main pathophysiological mechanism. The results of animal studies demonstrated myocardial hypertrophy and dysfunction with a hypercontractile state in both conditions of vitamin D deficiency as well as in VDR knockout models [23,30,81].

## 5. Guidelines

The Kidney Disease Improving Global Outcomes (KDIGO) CKD-MBD guidelines (2017) [82] suggest that in patients with CKD G3a–G5D, 25(OH)D (calcidiol) levels might be measured, and that vitamin D deficiency and insufficiency should be corrected using treatment strategies recommended for the general population (2C). Moreover, in patients with CKD G3a–G5 not on dialysis and with levels of intact PTH progressively rising or persistently above the upper normal limit for the assay, they recommend that PTH level ought to be evaluated for modifiable factors, including hyperphosphatemia, hypocalcaemia, high phosphate intake, and vitamin D deficiency (2C). According to these guidelines, in adult patients with CKD G3a–G5 not on dialysis, calcitriol and vitamin D analogues should not be routinely used (2C) since it is reasonable to reserve the use of calcitriol and vitamin D analogues for patients with CKD G4–G5 with severe and progressive hyperparathyroidism (Not Graded). However, in children, calcitriol and vitamin D analogues may be considered in order to maintain serum calcium levels in the age-appropriate normal range (Not Graded) [82].

## 6. Summary

Multiple observational studies have demonstrated an association between the use of active vitamin D therapy in patients on dialysis and with CKD and improved survival [55,57,83,84]. However, there are also many studies indicating important adverse effects of such treatment. Therefore, large randomized trials are required to analyse whether supplementation of vitamin D may affect outcomes and whether it is safe to be used in CKD patients.
